# Transferrin Receptor Binding BBB-Shuttle Facilitates Brain Delivery of Anti-Aβ-Affibodies

**DOI:** 10.1007/s11095-022-03282-2

**Published:** 2022-05-10

**Authors:** Rebecca Faresjö, Hanna Lindberg, Stefan Ståhl, John Löfblom, Stina Syvänen, Dag Sehlin

**Affiliations:** 1 Department of Public Health and Caring Sciences/Geriatrics, Uppsala University, Rudbeck Laboratory, Dag Hammarskjölds väg 20, 751 85 Uppsala, Sweden; 2 Department of Protein Science, School of Engineering Sciences in Chemistry, Biotechnology and Health, KTH Royal Institute of Technology, 106 91 Stockholm, Sweden

**Keywords:** affibody, transferrin receptor, transferrin receptor mediated transcytosis (RMT), protein brain delivery

## Abstract

Affibodies targeting amyloid-beta (Aβ) could potentially be used as therapeutic and diagnostic agents in Alzheimer’s disease (AD). Affibodies display suitable characteristics for imaging applications such as high stability and a short biological half-life. The aim of this study was to explore brain delivery and retention of Aβ protofibril-targeted affibodies in wild-type (WT) and AD transgenic mice and to evaluate their potential as imaging agents. Two affibodies, Z5 and Z1, were fused with the blood–brain barrier (BBB) shuttle single-chain variable fragment scFv8D3. *In vitro* binding of ^125^I-labeled affibodies with and without scFv8D3 was evaluated by ELISA and autoradiography. Brain uptake and retention of the affibodies at 2 h and 24 h post injection was studied *ex vivo* in WT and transgenic (tg-Swe and tg-ArcSwe) mice. At 2 h post injection, [^125^I]I-Z5 and [^125^I]I-Z1 displayed brain concentrations of 0.37 ± 0.09% and 0.46 ± 0.08% ID/g brain, respectively. [^125^I]I-scFv8D3-Z5 and [^125^I]I-scFv8D3-Z1 showed increased brain concentrations of 0.53 ± 0.16% and 1.20 ± 0.35%ID/g brain. At 24 h post injection, brain retention of [^125^I]I-Z1 and [^125^I]I-Z5 was low, while [^125^I]I-scFv8D3-Z1 and [^125^I]I-scFv8D3-Z5 showed moderate brain retention, with a tendency towards higher retention of [^125^I]I-scFv8D3-Z5 in AD transgenic mice. Nuclear track emulsion autoradiography showed greater parenchymal distribution of [^125^I]I-scFv8D3-Z5 and [^125^I]I-scFv8D3-Z1 compared with the affibodies without scFv8D3, but could not confirm specific affibody accumulation around Aβ deposits. Affibody-scFv8D3 fusions displayed increased brain and parenchymal delivery compared with the non-fused affibodies. However, fast brain washout and a suboptimal balance between Aβ and mTfR1 affinity resulted in low intrabrain retention around Aβ deposits.

**Supplementary Information:**

The online version contains supplementary material available at 10.1007/s11095-022-03282-2.

## INTRODUCTION

Biological drugs are growing rapidly on the pharmaceutical market, and are typically targeted towards peripheral diseases, such as cancer, rheumatism and autoimmune diseases. The major obstacle for developing biological drugs for central nervous system (CNS) diseases is their insufficient transport across the blood–brain barrier (BBB). Several of our studies using radiolabeled antibodies have shown that less than 0.05% of the administered dose resides in the brain 2 h after *i.v.* or *i.p.* injection (1–4). The BBB regulates the homeostasis of the CNS, and restricts the passage of molecules meticulously. Paracellular transport is controlled by tight-junctions between the brain capillary endothelial cells (BCECs), only allowing small (< 500 Da) lipophilic compounds to readily cross by this route. Biological drugs are typically large (> 1 kDa) hydrophilic molecules, and their passive transport across the BBB is therefore restricted. However, a possible route to reach the brain for a small fraction of macromolecules is via the blood-cerebrospinal fluid barrier (BCSFB) at the choroid plexus, and then through exchange between the cerebrospinal fluid (CSF) and the interstitial fluid (ISF) of the brain (5). In recent decades, active transport across the BBB has been explored as a strategy to increase brain delivery of biologics. The most studied method is the use of the endogenous receptor-mediated transcytosis (RMT) systems to facilitate increased transport of administered biologicals to the brain parenchyma. The transferrin receptor 1 (TfR1) is highly expressed on BCECs, and its endogenous function is to transport transferrin, carrying iron to the brain. The concept of using TfR1 as a “Trojan horse” to increase brain uptake of antibodies has been successful in many preclinical studies (1, 6–12). The mouse transferrin receptor (mTfR1) antibody 8D3-based BBB-shuttle is widely used for efficient brain delivery in preclinical studies in mice (1, 2, 13, 14). 8D3 binds to the apical domain of TfR1, thus not interfering with transferrin binding and the endogenous mechanism of the receptor (10).

Alzheimer’s disease (AD) is the most common form of dementia, and the number of cases is projected to triple by the year 2050 (15). To date, there are no disease modifying treatments for AD (16). Pathological hallmarks of AD are neurofibrillary tangles of aggregated tau protein and amyloid plaques, caused by the pathological aggregation of the protein amyloid-beta (Aβ). Aβ oligomers and protofibrils are pre-stages of amyloid plaques, and are assumed to exhibit toxicity already at pre-symptomatic stages of the disease (17, 18). Despite promising results in preclinical studies, numerous clinical trials with anti-Aβ antibodies have failed, possibly because of too late intervention, or that they were directed towards the wrong species of Aβ (19). While Aβ is still one of the main targets to halt disease progression, focus has shifted towards earlier detection and treatment (20). Several biotherapeuticals directed towards Aβ, including oligomeric and protofibrillar forms, in the pipeline are based on antibodies (21). Recently, the American Food and Drug Administration (FDA) conditionally approved the first immunotherapy for AD, an anti-Aβ antibody (Aduhelm®) against aggregated forms of Aβ (22).

Non-immunoglobulin therapeutic proteins are emerging as potential alternatives to conventional antibodies. Affibodies are antibody mimicking affinity proteins, originally based on an immunoglobulin G (IgG) binding domain of protein A in *Staphylococcus aureus* (23). The affibody molecule is based on a three alpha-helix scaffold of 6.5 kDa and is thus 20 times smaller than IgGs (24). The small molecular size, stability, robustness, as well as convenience of bacterial production and chemical synthesis are some of the advantages with this class of proteins. Affibodies also have several desirable properties for imaging. The small size, associated with fast systemic elimination, is required to achieve high-contrast *in vivo* imaging. Moreover, affibodies generally have higher tissue penetration and are more stable proteins compared with antibodies. Therefore, affibodies have been successful as radio-imaging probes in oncology and have progressed as radioligands into late clinical trials, e.g. for HER2 positive breast cancer (25–27).

Affibodies directed towards pathological proteins involved in neurodegenerative diseases, including α-synuclein, tau and Aβ, have also been developed (28–30). Z_SYM73_, is a high affinity (21 pM, 11.2 kDa) Aβ-monomer targeting affibody. Conjugated with an albumin-binding domain (ABD) to increase circulation time, Z_SYM73_-ABD, was able to both decrease the Aβ plaque load and reduce cognitive decline in an AD mouse model (28). This study points to affibodies’ potential as therapeutic agents for AD, and is also indicative of affibodies ability to exhibit pharmacological effects within the CNS. The distribution of affibodies to the brain parenchyma has not been studied in detail, but despite being smaller than IgGs, affibodies are still likely to be too large for efficient passive uptake at the BBB. Delivery to the CSF was investigated for Z_SYM73_-ABD and a Z_SYM73_-ABD fused with the antibody 8D3’s mTfR1-binding single chain variable fragment (scFv8D3). Increased concentrations in the CSF was shown for scFv8D3-Z_SYM73_-ABD after 24 h, whereas Z_SYM73_-ABD CSF-concentrations decreased over time, in correlation with its blood serum concentration profile (32). The study suggests that scFv8D3-Z_SYM73_-ABD was actively delivered to the CNS by TfR1 mediated uptake.

Small affinity proteins, like affibodies, directed toward oligomeric forms of Aβ could be developed into diagnostic agents for pre-symptomatic detection of AD pathology. There are currently no oligomer or protofibril-specific imaging probes used in clinic. We have previously developed preclinical positron emission tomography (PET) ligands based on antibodies, able to visualize intra-brain Aβ-pathology *in vivo* (33–36). A small (58 kDa) antibody-based fusion protein, di-scFv3D6-8D3, showed more favorable imaging pharmacokinetics compared to an IgG-like bispecific antibody (33, 37). However, biological circulation time of antibodies are typically too long to match the half-life of radioisotopes relevant for clinical usage (38). The aim of this study was to explore brain delivery and retention of Aβ-protofibril targeted affibodies in wild-type (WT) mice and two transgenic AD mouse models (tg-Swe and tg-ArcSwe), to evaluate their potential as PET imaging agents.

## MATERIALS AND METHODS

### Affibodies

The two affibody binders used in this study, Z_Aβ42cc_1_-His_6_ and Z_Aβ42cc_5_-His_6_ were generated toward protofibrils of Aβ_42_CC, an engineered mimic of wild-type Aβ_42_ protofibrils (described in ref. 39) (39). The affibodies displayed affinities in the low nanomolar range for Aβ_42_CC protofibrils (K_D_ = 1.7 ± 0.6 nM), determined by surface plasmon resonance, and also detected wild type Aβ_42_ protofibrils in ELISA (39).

In the present study, each of these two constructs were genetically fused to a BBB-shuttle, a single-chain fragment variable (scFv) of 8D3 (see “Cloning and protein production”). The four affibody constructs are hereafter referred to as Z5, scFv8D3-Z5, Z1 and scFv8D3-Z1 in this study (Table I). The measured molecular masses of Z5, scFv8D3-Z5, Z1 and scFv8D3-Z1 were in agreement with the expected theoretical values (Supplementary Fig. 1). However, the sample with scFv8D3-Z5 contained one additional protein of unknown origin, which we failed to remove in the purification (Supplementary Fig. 1). scFv8D3-Z5 was still included in the study with the assumption that the additional protein would not interfere with the functionality of scFv8D3-Z5, as binding to Aβ was retained indicated by Aβ protofibril ELISA. The aim was to study brain uptake and binding to Aβ in the brain of the affibodies and fused affibodies.

### Cloning and Production of Proteins

The genetic sequences for the scFv8D3-fusion proteins were cloned into the pQMCF1 vector (Icosagen Cell Factory OU, Tartu, Estonia) (40), containing a CMV promoter and an N-terminal CD33 signal peptide for secretion of the produced proteins. The 8D3 sequence was formatted into a single-chain fragment variable in the heavy to light chain direction, and connected by an 18 amino-acid glycine/serine-rich flexible linker (^N^SSGTTAASGSSGGSSSGA^C^). The 8D3 scFv was linked via a 10 amino-acid linker (^N^GAPGGGGSTS^C^) to the N-terminus of either of the amyloid beta protofibril-binding affibody molecules Z1 and Z5, respectively. Genetic sequences for control affibody molecules Z1 and Z5 were cloned into the pET26b( +) vector (Novagen), introducing a C-terminal His_6_-tag. The resulting plasmids (pQMCF1[scFv8D3-Z1-His_6_], pQMCF1[scFv8D3-Z5-His_6_], pET26b( +)[Z1-His_6_] and pET26b( +)[Z5-His_6_] were sequence verified by Sanger sequencing (Microsynth AG, Balgach, Switzerland).

pQMCF1 plasmids, containing the scFv8D3-fused proteins, were transfected into Chinese Hamster Ovary (CHO) EBNALT 85 cells for production using the Icosagen QMCF technology (40). Cells were cultivated for 13 days, followed by recovery of the fusion proteins from the cell culture supernatants. pET26b( +) vectors, containing the genes encoding non-scFv-fused proteins were transformed by heat shock into *Escherichia coli* BL21 Star (DE3) cells (Novagen, Madison, WI, USA) for production, according to preciously described protocol (41). Overnight-cultivated cells were harvested by centrifugation and lysed by sonication. All proteins were purified by immobilized metal affinity chromatography (IMAC) using a HisPur™ Cobalt resin (Thermo Fisher Scientific, Rockford, USA) under native conditions. Eluted proteins were buffer-exchanged to PBS using PD-10 desalting columns (GE Healthcare Life Sciences, Uppsala, Sweden). The protein concentration was determined by absorbance measurement at 280 nm. The molecular weight and purity of the purified proteins were subsequently confirmed using SDS-PAGE (NuPAGE™ 4–12% Bis–Tris gels) and MALDI mass spectrometry (4800 MALDI-TOF).

### Radiochemistry

All four affibodies were radiolabeled by direct iodination using the chloramine T method (42). Briefly, 20–40 μg affibody was mixed with ^125^I stock solution (Perkin-Elmer Inc Waltham, MA, USA). Thereafter, 5 μg Chloramine-T (Sigma Aldrich, Stockholm, Sweden) in PBS was added to a final volume of 110 μl. The reaction was incubated for 90 s at room temperature, and quenched with 10 μg sodium metabisulfite (Sigma Aldrich). The product was diluted to 500 μl with PBS, and separated from free-iodine in a PBS-equilibrated NAP-5 size exclusion column (Cytiva, Uppsala, Sweden). The product was eluted with a total volume of 1 ml PBS.

Pierce Pre-Coated Iodination Tubes (ThermoFischer, Rockford, IL, USA) was used as an alternative to the Chloramine T method to achieve milder reaction conditions (43). First, 1 ml PBS was used to wet the iodination tube. ^125^I stock solution (Perkin-Elmer) was added together with PBS to the iodination tube to a total volume of 40 μl. The tube was incubated for 6 min on a shaker at room temperature to activate the iodine. 10–20 μg affibody was prepared in Protein LoBind tubes (Eppendorf). Thereafter, 20 μl activated iodine was added to each affibody, followed by a 10 min incubation at room temperature on a shaker (600 rpm). After the incubation, the product was diluted with PBS to 500 μl and purified as described above.

### *In Vitro* Validation of Radiolabeled Affibodies

The affibody binding to Aβ protofibrils and mTfR1 before and after radiolabeling was assessed with indirect ELISA in comparison with di-scFv3D6-8D3 (37). In short, 96-well half area plates (Corning Inc.) were coated with 250 nM Aβ-PF (Innovagen) in PBS or 2 µg/ml mTfR1 (BioArctic, Stockholm, Sweden) in PBS and incubated at 4°C overnight. The plates were blocked with 1% BSA in PBS. The affibodies were serially diluted from 250 nM (and di-scFv3D6-8D3 from 50 nM) and incubated overnight at 4°C. The Aβ-PF plates were incubated for 1 h with HRP-conjugated anti-His-Tag antibody (Proteintech Goup INC., IL, USA), while mTfR1 plates were incubated first for 1 h with Goat-Anti-Affibody IgG (Affibody AB, Stockholm, Sweden) followed by 1 h incubation with Rabbit-Anti-Goat-HRP (ThermoFischer). Signals were developed with K Blue Aqueous TMB substrate (Neogen Corp., Lexington, KY, USA) and the reaction stopped after 10 min with 1 M H_2_SO_4_. The plates were analyzed at 450 nm in a spectrophotometer. Affibody and secondary antibody sample dilutions were made in ELISA incubation buffer (PBS, 0.1% BSA, 0.05% Tween-20).

Sandwich ELISA was used to determine concentrations of affibodies after radiolabeling. Half area 96-well plates (Corning Inc.) coated with 0.5 μg/ml Goat-Anti-Affibody IgG (Affibody AB, Stockholm, Sweden) were incubated overnight in at 4°C. After 1 h of blocking with 1% BSA in PBS, affibody samples were serially diluted from 250 nM, followed by incubation overnight at 4°C. The plates were incubated with HRP-conjugated anti-His-Tag antibody (Proteintech) for 1 h incubation on a shaker. The signal was developed and plates read as described above.

### Animals

Two AβPP transgenic mouse models were used in this study to investigate the *in vivo* retention of the affibody constructs: tg-Swe, harbouring the Swedish (APP KM670/671NL) APP mutation, and tg-ArcSwe with a combination of the Arctic (APP E693G) and the Swedish APP mutations (tg-ArcSwe), both maintained on a C57BL/6 background. The mutation in tg-Swe mice leads to increased production of Aβ, and a late onset (at 12 months) of Aβ pathology, followed by rapid progression. In tg-ArcSwe mice, increased Aβ production in combination with an aggregation-prone Aβ mutated species produces an earlier onset (at 6 months) of pathology with dense Aβ-plaques (44). These two models are suitable to study the variation in Aβ pathology, from plaques to soluble oligomers. Another reason to use both models was that tg-ArcSwe produces a mutated form of Aβ (Arctic mutation, within the Aβ sequence), while tg-Swe only produces wild-type Aβ (Swedish mutation, outside the Aβ sequence). C57BL/6 WT mice were used as control animals and to study brain delivery of the proteins. All animals were between 17–24 months old and both males and females (n = 67, f = 44 m = 19) were used for the experiments. The animals were housed in an approved animal facility at the Uppsala University with *ad libitum* access to food and water. All described procedures were approved by the Uppsala Country Animal Ethics board (5.8.18–13,350/17) following the legislation and regulations of the Swedish Animal Welfare Agency and European Communities Council Directive of 22 September 2010 (2010/63/EU).

### *In Vitro* Autoradiography

Cryosections, 20 μm, were prepared from brains of old (2 years) wild type and tg-ArcSwe mice and mounted on Superfrost Plus glass slides (Menzel Gmboltion, Braunschweigh, Germany). The frozen sections were adjusted to room temperature for 1 h, and blocked with 1% BSA in PBS for 1 h. The sections were incubated in PBS for 5 min, followed by the addition 4 nM of [^125^I]I-Z5 or [^125^I]I-Z1 (0.9 MBq/nmol), and 1 nM of [^125^I]I-scFv8D3-Z5 or [^125^I]I-scFv8D3-Z1 (3–4 MBq/nmol) in PBS for overnight incubation at 4°C. The sections were washed 3 × 15 min in cold 0.1% BSA-PBS-buffer, 20 s in dH_2_O, and dried under constant air flow at RT for 1 h, then exposed to a phosphor imaging plate (MS, MultiSensitive, PerkinElmer, Downers Grove, IL, USA) for 3 h. The plate was scanned and digitalized at 600 dpi in a Cyclone Plus phosphor imager (PerkinElmer). Images were converted with the “Royal” lookup table and the intensity was adjusted individually for each protein in ImageJ. Regions of interest (ROIs) were quantified using the Integrated Density (IntDen) measurement in triplicate brain sections.

### *Ex Vivo* Study ^125^I-Labeled Affibodies

Mice were intravenously injected with ^125^I-labeled affibodies via the tail vein. The animals were injected with either 0.49 ± 0.20 MBq of [^125^I]I-Z5, 0.43 ± 0.18 MBq of [^125^I]I-scFv8D3-Z5, 0.76 MBq ± 0.13 MBq of [^125^I]I-Z1 or 0.88 ± 0.34 MBq of [^125^I]I-scFv8D3-Z1. To investigate the blood pharmacokinetic profiles, blood samples of 8 μl were collected from the tail vein at time points between 30 min after administration and euthanization, i.e. at 2 h or 24 h post injection. At euthanization, transcardial perfusion under isoflurane anesthesia with 40 ml saline for 2.5 min was used to remove blood from the brain and organs before isolation. Subsequently, the brain was harvested and dissected into the right hemisphere, left cerebrum and left cerebellum. The brain samples were immediately frozen at -80°C. Lung, liver, kidney, heart, pancreas, spleen, femoral bone, skull bone and submandibular gland (smg) were isolated to study the biodistribution of the radiolabeled affibodies. The radioactivity of all samples was measured with a γ-counter (2480 Wizard™, Wallac Oy PerkinElmer, Turku, Finland). Antibody concentrations were expressed as percent of injected dose per gram tissue (%ID/g) or percent of injected dose corrected for body weight (bw) of the animal (%ID/g/bw). The reported brain concentrations were measured in the cerebrum of the left hemisphere.

### *Ex Vivo* Autoradiography

The frozen right hemispheres from [^125^I]I-affibody-injected animals were sectioned sagittally (20 μm) with a cryostat (CM1850, Leica Biosystems, Nussloch, Germany) and mounted on Superfrost Plus glass slides (Menzel Gmboltion). Duplicate sections from each animal together with a standard of ^125^I with known radioactivity were exposed to a phosphor imaging plate (MS, Multisensitive, PerkinElmer, Downers Grove, IL, USA) for 7 days. The plates were scanned in a Cyclone Plus phosphor imager (PerkinElmer) at 600 dots per inch.

### Immunofluorescence Staining

Sagittal brain cryosections from [^125^I]I-affibody-injected mice were fixed in ice-cold MeOH for 10 min and washed 2 × 5 min in PBS. Double CD31/Aβ40 staining was performed on selected WT, tg-Swe and tg-ArcSwe brain sections by the following procedure:

The sections were blocked for 1 h with 5% Normal Goat Serum, followed by a wash in PBS. The primary antibodies rat-α-mouse CD31 (BD, #553,370) and rabbit-α-Aβ40 (Agrisera, Umeå, Sweden) or 6E10 (Nordic Biosite, Täby, Sweden) for di-scFv3D6-8D3, was applied to the sections which were then incubated overnight at 4°C with slow shaking. After incubation, the sections were washed in PBS and secondary antibody goat-α-rat (Alexa 488) and goat-α-rabbit (Alexa 647) was added for 1 h with slow shaking, followed by a PBS wash. The sections were stored in PBS until the nuclear track emulsion procedure (described below) was performed on the same day.

Neuronal marker, rabbit-anti-mouse NeuN (Abcam, ab177487, Cambridge, UK), and Aβ staining with 6E10 (Nordic Biosite) was used for the brain section to illustrate pathology in Fig. 2b.

### Nuclear Track Emulsion Autoradiography

Nuclear track emulsion autoradiography experiments were done in darkness as previously described (6). In brief, ILFORD K5 emulsion was prepared in a 40°C water bath according to manufacturer’s instructions. The immunofluorenscently stained sections were immersed in the emulsion for 5 s and left to air dry for 2 h, then incubated in darkness for 4 weeks at 4°C. The sections were developed according to the manufacturers’ instructions and dehydrated in increasing EtOH concentration gradient (70%, 95%, 100%) and mounted with Pertex (Histolab). Images of the developed emulsion and CD31-immunofluorescent stained sections were acquired with a Zeiss Observer Z.1 microscope (Carl Zeiss Microimaging GmbH, Jena, Germany) and processed equally using the ZEN software. An inverted lookup table was applied to the brightfield channel, resulting in white emulsion puncta instead of black.

### Statistical Analysis

Data is presented as mean ± standard deviation, if not stated otherwise. All calculations were done in Prism v. 9.2.0 (GraphPad Software, Inc.). Saturation binding curves were used to fit the ELISA data and estimate the K_D_ values for the affibodies. One-way analysis of variance with Bonferroni correction was used to compare 2 h brain uptake of the affibodies. Quantification of *in vitro* autoradiography sections and 24 h brain retention were analyzed with unpaired t-tests to compare tg-ArcSwe and WT brain sections.

## Results

### *In Vitro* Analyses of Affibodies

The four affibody proteins were initially assessed for binding to Aβ protofibrils and mTfR1 *in vitro*. The fusion antibody di-scFv3D6-8D3 was used for comparison (33).

**Fig. 1:**
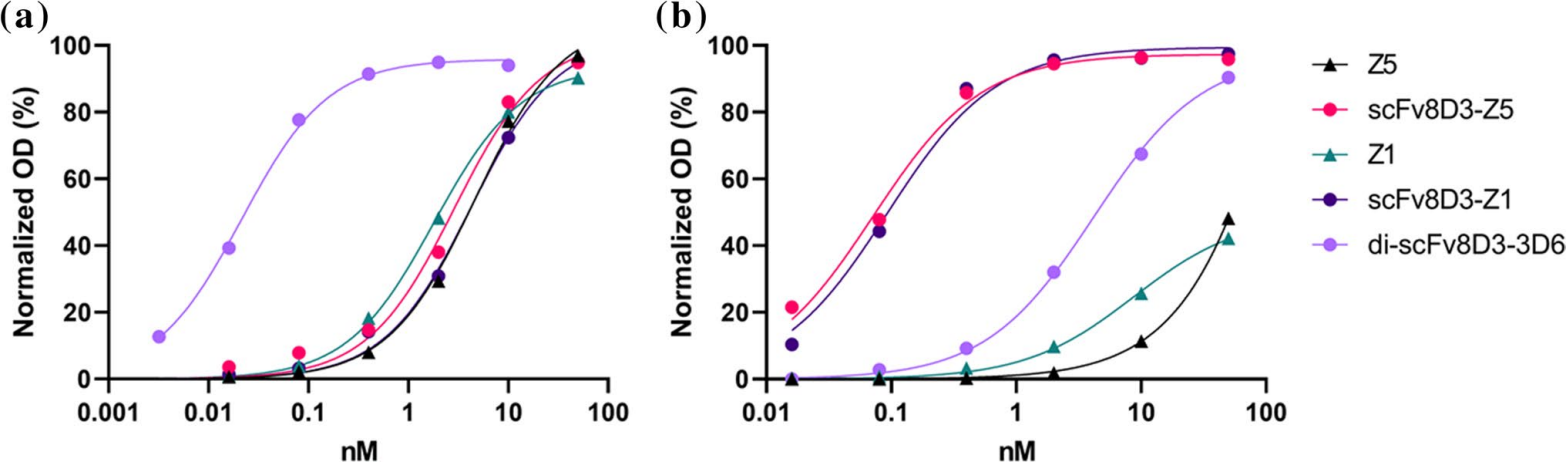
Indirect ELISA of the four affibodies and bispecific Aβ-antibody fusion protein di-scFv3D6-8D3. Plates were coated with (**a**) 250 nM Aβ protofibrils (**b**) 2 μg/ml mTfR1.

Aβ protofibril ELISA indicated that the affibodies bound Aβ protofibrils with a 12–35 nM affinity. The fused scFv8D3-Z5 retained binding in the same range after conjugation to scFv8D3, but scFv8D3-Z1 displayed increased K_D_ after conjugation (Fig. 1, Table II). The affibodies showed more than 100-fold lower affinity towards Aβ protofibrils compared with di-scFv3D6-8D3 (Fig. 1a). As expected, the mTfR1 binding was higher for the scFv8D3-fused affibodies, compared with Z5 and Z1, which displayed some background binding at high concentrations. Surprisingly, the scFv8D3-fused affibodies displayed at least 20-fold higher binding in the mTfR1 assay compared to di-scFv3D6-8D3 (Fig. 1b).

**Table I:** Affibody Protein, Shortened Name and the Respective Molecular Weight

Protein	Short name	Mw (kDa)
Z_**Aβ42cc_5**_-His6	Z5	7.6
Z_**Aβ42cc_1**_-His6	Z1	7.5
scFv8D3-linker-Z _**Aβ42cc_5**_-His6	scFv8D3-Z5	34.8
scFv8D3-linker-Z_**Aβ42cc_1**_-His6	scFv8D3-Z1	34.7

**Table II:** Affinities (K_D_) in nM for Aβ-Protofibrils and mTfR1 Determined by Indirect ELISA for the Affibodies and di-scFv3D6-8D3. Mean Fold Difference in K_D_ Between Unlabeled and ^125^I-Labeled Affibody and di-scFv3D6-8D3, Towards Aβ-protofibrils and mTfR1 Determined by Indirect ELISA (Values Expressed as Mean ± SD)

Protein	Aβ-protofibrils (nM)	Fold difference after radiolabeling	mTfR1 (nM)	Fold difference after radiolabeling
Z5	13.1 ± 7.1	1.4 ± 0.4	n/a	n/a
scFv8D3-Z5	11.8 ± 6.4	1.4 ± 0.3	0.05 ± 0.02	2.0 ± 0.4
Z1	12.4 ± 9.8	2.0 ± 0.5	n/a	n/a
scFv8D3-Z1	35.3 ± 37.3	1.1 ± 0.1	0.10 ± 0.02	3.0 ± 0.2
di-scFv3D6-8D3	0.08 ± 0.04	1.5 ± 0.3	2.1 ± 1.7	2.0 ± 1.3

Radiolabeled affibodies were evaluated by ELISA after each labeling. Binding towards Aβ protofibril and mTfR1 was in general retained for all of the affibodies, but with a minor increase in the K_D_ after radiolabeling. The average difference in K_D_ towards Aβ protofibrils and TfR1 between unlabeled and ^125^I-labeled affibody is shown in Table II.

### *In Vitro* Autoradiography

**Fig. 2:**
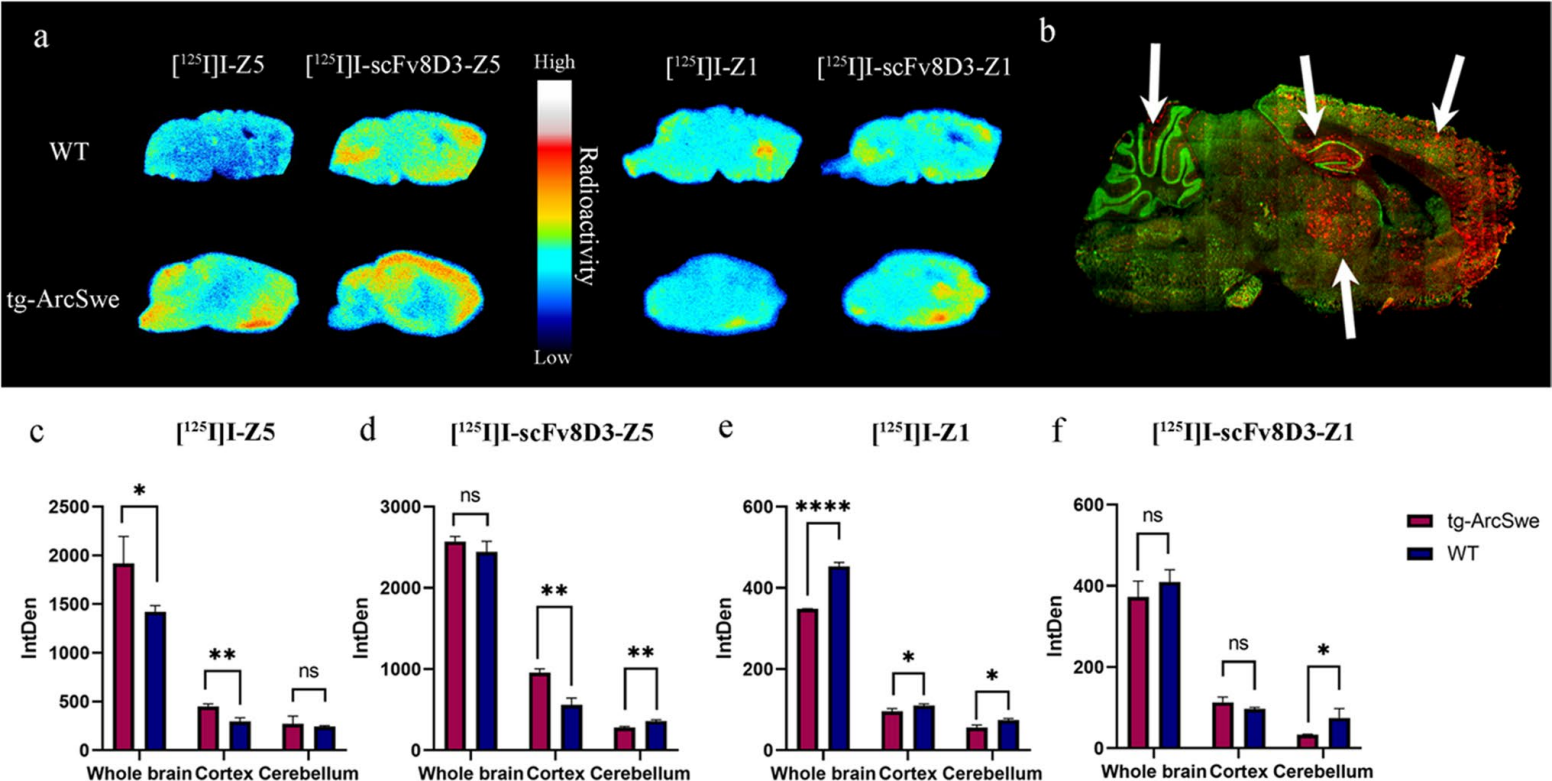
(**a**) *In vitro* autoradiography of [^125^I]I-Z5, [^125^I]I-scFv8D3-Z5, [^125^I]I-Z1 and [^125^I]I-scFv8D3-Z1, applied to sagittal brain sections of old WT and tg-ArcSwe mice. (N.B. different scales were used for each protein) (**b**) Pathology visualized by fluorescent Aβ staining (red) in a tg-ArcSwe sagittal mouse brain section. White arrows indicate areas of pathology (top row from left to right); cerebellum, hippocampus, cortex; (bottom row); thalamus. Neuronal marker (NeuN) is shown in green. (**c**-**f**) Quantification of *in vitro* autoradiography sections and unpaired t-test to compare tg-ArcSwe and WT sections (* *p* ≤ 0.05, ** *p* ≤ 0.01, *** *p* ≤ 0.001, **** *p* ≤ 0.0001).

*In vitro* autoradiography showed that brain sections exposed to [^125^I]I-Z5 displayed higher binding in the cortex of tg-ArcSwe, compared with WT mouse brains (Fig. 2a and c). Aβ pathology related binding was even more pronounced in tg-ArcSwe sections exposed to [^125^I]I-scFv8D3-Z5 (Fig. 2a and d). Compared to WT sections, the [^125^I]I-scFv8D3-Z5 signal appeared higher in cortex, hippocampus and thalamus (Fig. 2a), regions typically associated with pathology in the tg-ArcSwe mouse (Fig. 2b). In the cerebellum (a region largely devoid of pathology) the binding was lower and comparable with WT. [^125^I]I-Z1 and [^125^I]I-scFv8D3-Z1 had generally lower binding compared to [^125^I]I-Z5 and [^125^I]I-scFv8D3-Z5 sections (Fig. 2a). Interestingly whole brain signal was higher in WT compared to tg-ArcSwe for [^125^I]I-Z1, but neither [^125^I]I-Z1 nor [^125^I]I-scFv8D3-Z1 appeared to show increased binding in cortex, hippocampus or thalamus on the tg-ArcSwe sections (Fig. 2e-f), suggesting limited pathology-related binding of these iodinated affibodies.

### Peripheral Distribution of ^125^I-Labeled Affibodies

**Fig. 3:**
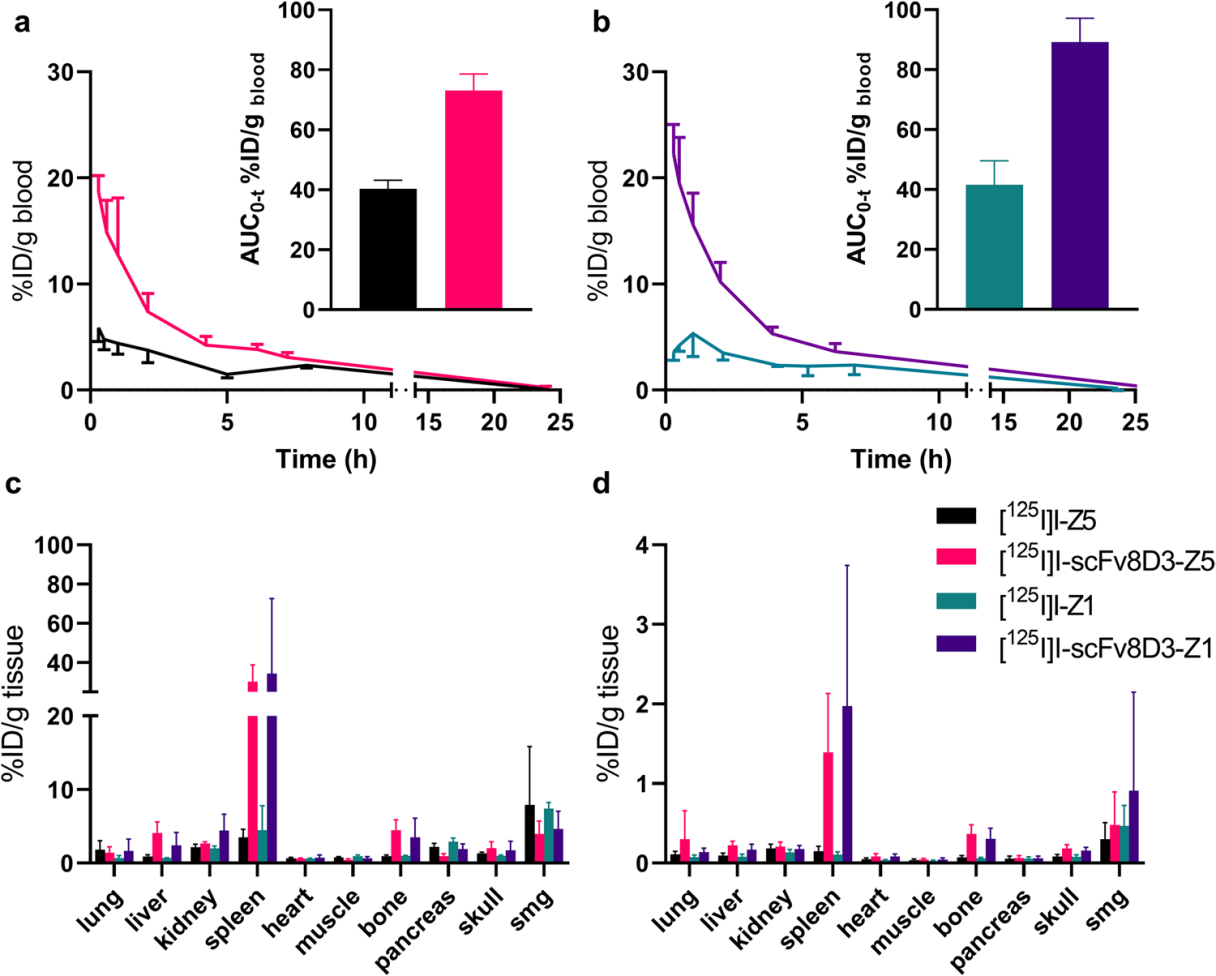
Whole blood time-concentration profiles and total exposure (AUC_0-t_) for (**a**) [^125^I]I-Z5 and [^125^I]I-scFv8D3-Z5 and (**b**) [^125^I]I-Z1 and [^125^I]I-scFv8D3-Z1, expressed as percentage of injected dose per gram blood (%ID/g). The lines represent mean ± SD. Peripheral biodistribution of [^125^I]I-Z5, [^125^I]I-scFv8D3-Z5, [^125^I]I-Z1 and [^125^I]I-scFv8D3-Z1, expressed as %ID/g tissue at (**c**) 2 h post injection and (**d**) 24 h post injection. (N.B. different scales on the y-axes for c and d), smg = submandibular gland.

Whole blood concentration–time curves showed fast blood clearance of [^125^I]I-Z5 and [^125^I]I-Z1, while scFv8D3-fused affibodies, [^125^I]I-scFv8D3-Z5 and [^125^I]I-scFv8D3-Z1, displayed increased circulation-time in blood (Fig. 3). The total exposure (area under the curve, AUC_0-t_) was about 2 times higher for the scFv8D3-fused affibodies compared with the non-fused affibodies (Fig. 3a-b). For [^125^I]I-Z1, blood radioactivity concentration appeared to reach maximum at 1 h post injection rather than immediately following injection.

At 2 h after injection, the distribution to peripheral organs differed mainly by a high distribution to spleen for the scFv8D3-fused affibodies. They also displayed a high uptake in the femoral bone, likely in the bone marrow (Fig. 3c). The difference remained noticeable also at 24 h post injection (Fig. 3d).

### Brain Uptake 2 h After Administration of ^125^I-Labeled Affibodies

**Fig. 4:**
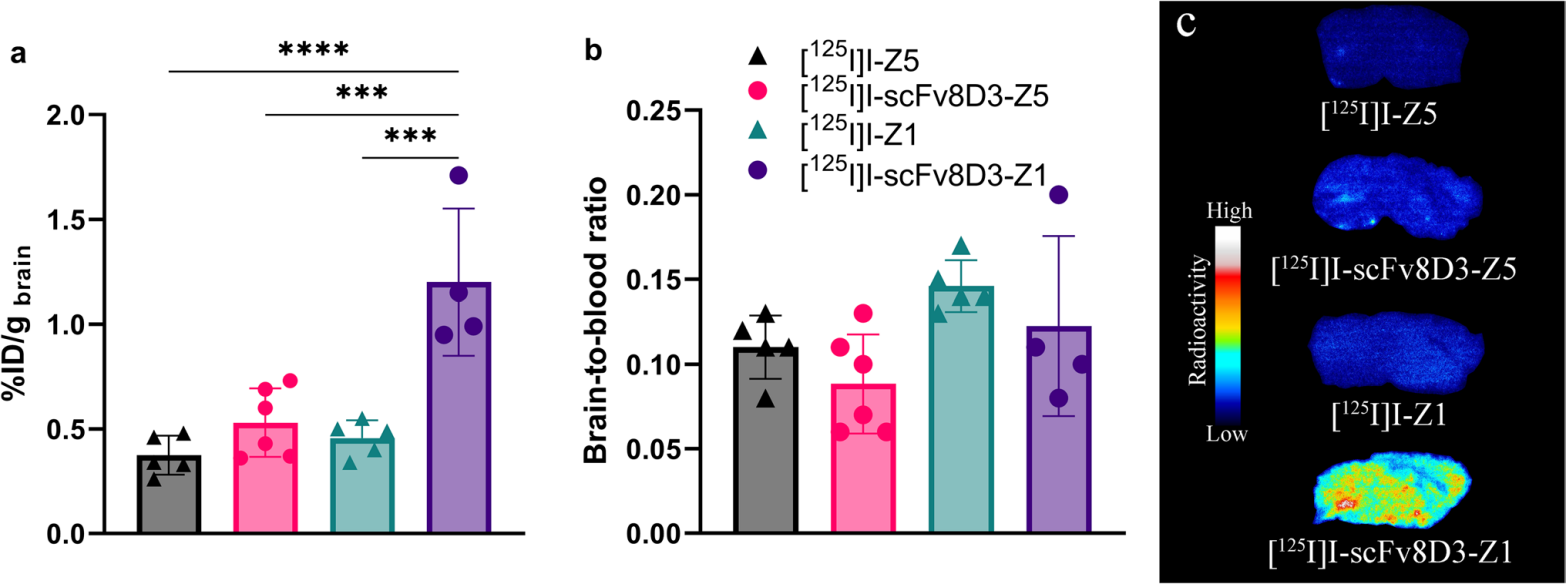
(**a**) Brain uptake 2 h after administration of [^125^I]I-Z5, [^125^I]I-scFv8D3-Z5, [^125^I]I-Z1 or [^125^I]I-scFv8D3-Z1 expressed as %ID/g brain. One-way analysis of variance with Bonferroni correction indicated significantly increased brain concentrations for [^125^I]I-scFv8D3-Z1 (**b**) Brain-to-blood ratio 2 h post injection. (**c**) E*x vivo* autoradiography illustrating the brain uptake after 2 h of representative sagittal brain sections from WT mice injected with [^125^I]I-Z5, [^125^I]I-scFv8D3-Z5, [^125^I]I-Z1 or [^125^I]I-scFv8D3-Z1.

All of the ^125^I-labeled affibodies showed a 10- to 20-fold higher total brain uptake at 2 h post injection than previously seen with ^125^I-radiolabeled IgG (1–3). [^125^I]I-Z5 had an average brain radioactivity concentration of 0.37% ± 0.09%ID/g_brain_, while [^125^I]I-Z1 had 0.46% ± 0.08%ID/g_brain._ Fusion with scFv8D3 further increased affibody brain concentrations to 0.53 ± 0.16%ID/g_brain_ for [^125^I]I-scFv8D3-Z5 and [^125^I]I-scFv8D3-Z1 had significantly increased brain concentrations of 1.20 ± 0.35%ID/g_brain_ (Fig. 4a). Thus, the increase from non-fused affibody was 43% for [^125^I]I-scFv8D3-Z5 and 161% for [^125^I]I-scFv8D3-Z1, with an average brain concentration of 0.80%ID/g brain for both fused affibodies. Thus, the scFv8D3-fused affibodies displayed brain concentrations comparable to other monovalent mTfR1-targeted bispecific antibodies in brain after 2 h (37). There was a trend towards slightly higher brain-to-blood ratios for Z5 and Z1 compared with their scFv8D3-fused versions, due to faster elimination from blood (Fig. 4c). *Ex vivo* autoradiography of brain sections from [^125^I]I-affibody injected mice visualized the differences in brain radioactivity 2 h post injection (Fig. 4c).

### Brain Retention 24 h After Administration of ^125^I-Labeled Affibodies

**Fig. 5:**
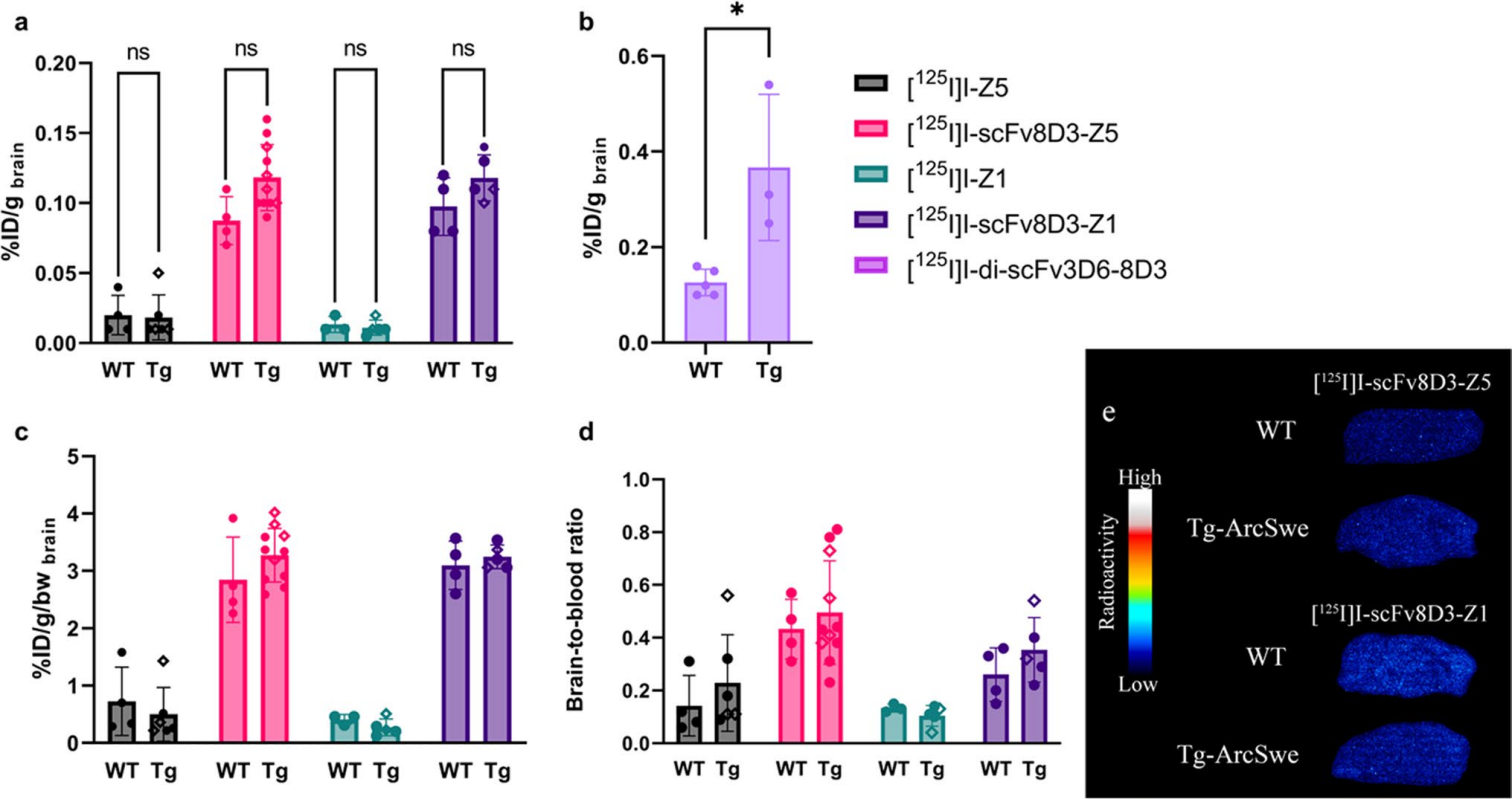
Brain retention at 24 h in wild-type (WT) and transgenic (Tg; tg-Swe ● or tg-ArcSwe ♦) mice (a) %ID/g brain for affibodies (**b**) %ID/g brain for [^125^I]I-di-scFv3D6-8D3 injected animals 24 h after administration. Unpaired t-tests showed significant difference between transgenic and WT in animals administered iodinated di-scFv3D6-8D3, but not for affibodies (**c**) %ID/g brain corrected for body weight (bw) for animals injected with affibodies (**d**) Brain-to-blood ratio 24 h post injection of affibodies. (**e**) *Ex vivo* autoradiography illustrating the radioactivity in selected sagittal mouse brain sections 24 h after injection with [^125^I]I-scFv8D3-Z5 or [^125^I]I-scFv8D3-Z1.

Brain retention of the iodinated affibodies in WT and transgenic mice was evaluated at 24 h after administration, to assess potential interactions with Aβ pathology. Affibodies lacking scFv8D3, [^125^I]I-Z5 and [^125^I]I-Z1, showed in general low retention in the brain at this time point, with no difference between WT and transgenic mice (Fig. 5a). Affibodies fused to scFv8D3 showed higher brain retention overall, and [^125^I]I-scFv8D3-Z5, in particular, showed a tendency to differentiate between WT and transgenic mice (Fig. 5a). However, this difference did not fully reach statistical significance (p = 0.06). The two fused affibodies displayed brain retention similar to what was observed in WT mice 24 h after injection of [^125^I]I-di-scFv3D6-8D3. In contrast, a significant difference between transgenic and WT animals was observed with [^125^I]I-di-scFv3D6-8D3, which indicates Aβ-specific retention (Fig. 5b). Correcting for animal body weight did not change the result between WT and transgenic groups for [^125^I]I-scFv8D3-Z5 (Fig. 5c). Notably, the brain-to-blood ratio was higher for the scFv8D3-fused affibodies, indicating a specific interaction in the brain (Fig. 5d).

*Ex vivo* autoradiography of sagittal brain sections from [^125^I]I-affibody-injected mice showed in general very low radioactivity and confirmed the results presented in Fig. 5a. [^125^I]I-Z5 and [^125^I]I-Z1-injected mice had no detectable signal after 24 h, while scFv8D3-fused affibodies displayed a weak radioactive signal (Fig. 5e). However, the signals were too low to detect any differences between WT and transgenic animals, or to reveal pathology related binding patterns (Fig. 5e).

### Nuclear Track Emulsion

**Fig. 6:**
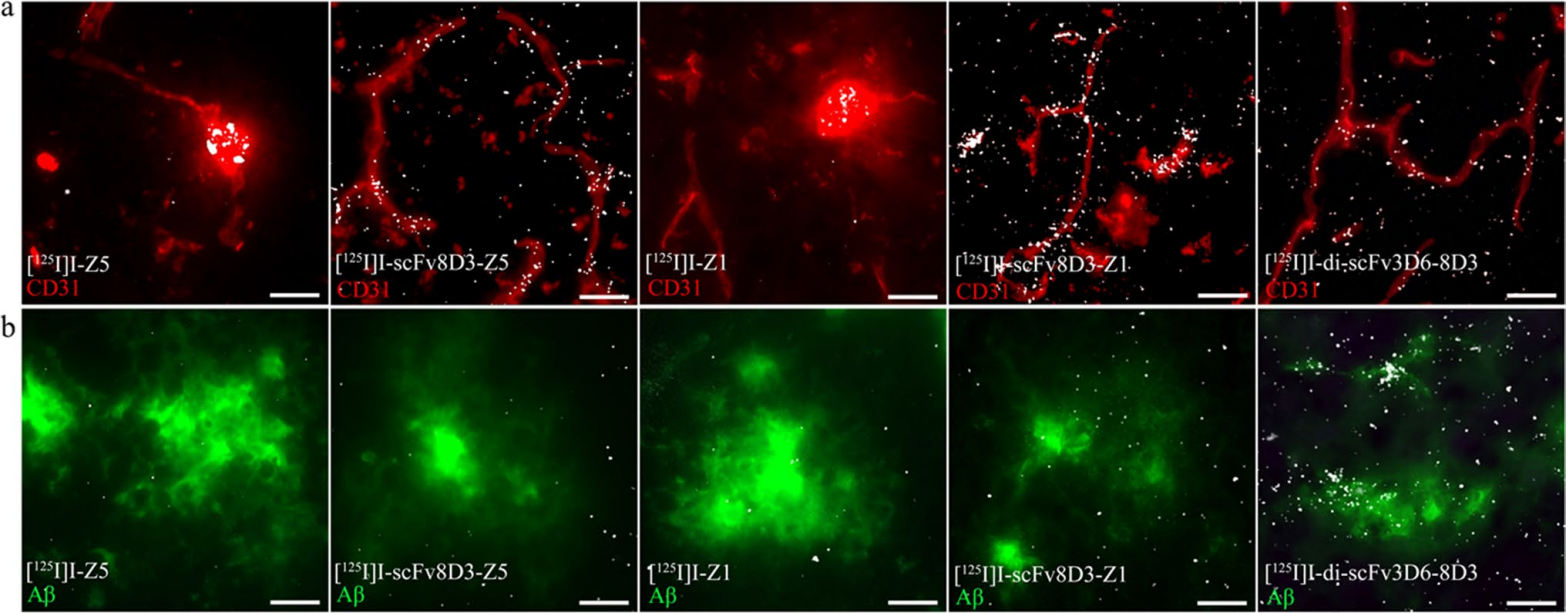
Representative images of nuclear track emulsion autoradiography illustrating the brain radioactivity (white) in sagittal brain sections from mice injected with [^125^I]I-Z5, [^125^I]I-scFv8D3-Z5, [^125^I]I-Z1, [^125^I]I-scFv8D3-Z1 or [^125^I]I-di-scFv3D6-8D3 (**a**) 2 h post-injection with staining of vascular marker CD31 (red) (**b**) 24 h post-injection and (12 h post-injection for [^125^I]I-di-scFv3D6-8D3) with Aβ (green) staining. Scale bar = 20 μm.

Nuclear track emulsion autoradiography, in combination with immunostaining of the vasculature (CD31) and Aβ, was used to study in detail how the affibodies distributed in the brain tissue. At 2 h post-injection, there was a clear difference in distribution of the non-fused and fused affibodies. The non-fused affibodies [^125^I]I-Z5 and [^125^I]I-Z1 appeared clustered, mainly in larger CD31-positive structures, with very little distribution to the parenchymal space and smaller capillaries (Fig. 6a). On the other hand, the signal from [^125^I]I-scFv8D3-Z5 and [^125^I]I-scFv8D3-Z1 was more scattered and highly distributed both to the brain capillaries and in parenchymal areas, similar to the distribution of di-scFv3D6-8D3, 2 h after injection (Fig. 6a).

At 24 h after injection, the affibody-derived signal was overall low, with no or little accumulation at Aβ-plaques for all four affibodies (Fig. 6b). For comparison, [^125^I]I-di-scFv3D6-8D3 displayed a high signal and visibly accumulated near Aβ-plaques (green) already 12 h post injection (Fig. 6b).

## DISCUSSION

In this study, we have explored Aβ protofibril targeted affibodies, and the possibility to enhance their brain uptake by TfR1 mediated transport across the BBB, and further, to evaluate them as potential brain-PET radioligands for *in vivo* imaging of Aβ. Affibodies Z5 and Z1 showed *in vitro* affinities for Aβ protofibrils in ELISA in the low nanomolar range (12–35 nM), i.e. in a similar range as previously reported (39). The binding to Aβ was largely retained after conjugation to the mTfR1-binding fragment scFv8D3 and after radioiodination.

For diagnostic imaging purposes, high brain uptake in combination with a fast clearance from blood is advantageous as a high brain-to-blood ratio is required. A small size could therefore be an important feature for an imaging radioligand, as it seems to govern the molecule’s residence time in blood (34). A short biological half-life could allow same-day PET imaging after labelling with fluorine-18, which decays with a half-life of 110 min. However, the blood half-life of singular affibody proteins in rats is only around 10–20 min (45). Since brain and blood concentrations are in equilibrium, this may be too short to achieve brain concentrations high enough for imaging. Here we observed a fast clearance of the non-fused affibodies [^125^I]I-Z1 and [^125^I]I-Z5, with low blood concentrations already 30 min after injection. For [^125^I]I-Z1 we observed a blood activity peak around 1 h after injection, likely attributed to metabolism and release of free iodine, which would suggest that blood concentrations of this affibody may be overestimated. Thus, the increased blood circulation time and AUC_0-t_ observed for the scFv8D3-fused affibodies in this study may be an advantage.

The radioiodinated affibodies, [^125^I]I-Z1 and [^125^I]I-Z5 showed surprisingly high brain uptake at 2 h after administration (0.37% and 0.46%ID/g brain, respectively), despite fast blood elimination. This is more than tenfold higher than brain concentrations previously observed for iodinated IgG antibodies (0.03%ID/g brain) (1–4, 46). These measurements are based on perfused bulk brain measurements, i.e. including the vascular compartment but not the blood of the brain. Indeed, the affibodies were highly associated with large vessels, but had low signal in parenchymal areas of the brain 2 h after injection. The affibody has a six histidine residue (6xHistag) affinity tag, which increases the net surface charge of the molecule. The high association to brain at the early time point could potentially be explained by interactions between the positively charged 6xHis tag and the negatively charged glycocalyx of the BBB endothelial cells. We have previously observed increased brain concentrations of a cationized Aβ protofibril selective F(ab')_2_ fragment by adsorptive transcytosis (AMT), which is slower and less specific than RMT (11, 47, 48).

BBB-shuttles such as scFv8D3 have proven important to potentiate the delivery of biologicals to the brain (1, 9, 10, 12, 33, 35, 49). Despite the relatively high apparent brain uptake of the non-fused affibodies, the addition of scFv8D3 increased total brain concentrations with 43% and 161% for Z5 and Z1, respectively, 2 h after administration. [^125^I]I-scFv8D3-Z1 had significantly higher brain concentration compared to the other affibodies. The lower initial brain concentrations of [^125^I]I-scFv8D3-Z5 compared with [^125^I]I-scFv8D3-Z1, could be due to lower purity of scFv8D-Z5 (Supplementary Fig. 1). We speculate that the impurity decreased the fraction of TfR1-binding protein at the BBB, leading to an overestimation of the injected dose of [^125^I]I-scFv8D3-Z5, and thus a lower apparent brain concentration. Despite this, we decided to include all [^125^I]I-scFv8D3-Z5 data, since little is known about intra-brain affibodies and their binding to Aβ in the brain. Moreover, both scFv8D3-fused affibodies showed increased brain uptake and parenchymal delivery compared to their non-fused versions. On average, they showed similar brain concentrations and parenchymal penetrance as the previously studied bispecific antibody construct di-scFv3D6-8D3 (58 kDa).

This confirms that TfR1 mediated transport is a robust strategy for enhancing brain delivery of affibody molecules (37). Di-scFv3D6-8D3 binds monovalently to mTfR1 (50), which has been reported to increase brain uptake (10, 51). We have previously shown that di-scFv3D6-8D3 displayed higher brain parenchyma-to-capillary ratio compared to the 210 kDa bivalent IgG-based antibody, mAb3D6-scFv8D3, potentially due to the monovalent TfR1 interaction of di-scFv3D6-8D3 (37). The impact of TfR1 affinity on therapeutic antibodies has also been studied, where a moderate affinity of 50 nM-100 nM was found optimal for brain delivery (52). Here, we found that although fused to a similar scFv8D3 fragment for monovalent TfR1 binding and BBB transport, the scFv8D3-fused affibodies displayed a markedly higher TfR1 affinity, compared with di-scFv3D6-8D3. This could potentially be due to a different folding of the protein, or a slight difference in linker design. The higher affinity would likely increase their ability to bind endothelial TfR1 at the BBB, but could also affect their ability to be released into the brain parenchyma and engage with an intrabrain target. The fused affibodies displayed a high spleen uptake compared with the non-fused affibodies. We have observed this previously for other proteins fused with scFv8D3 (53). The spleen uptake is probably related to mTfR1 interactions with cells of the spleen, and the levels observed in this study are indicative of a strong *in vivo* interaction due the high TfR1 affinity.

Nuclear track emulsion autoradiography showed that 2 h after administration, the scFv8D3-fused affibodies were highly distributed to endothelial cells in brain capillaries, but also to the parenchyma. The non-fused affibodies, however, seemed to cluster in larger vessels. Importantly, at 24 h after injection, although moderate, the brain retention of the scFv8D3-fused affibody molecules was considerably higher than for [^125^I]I-Z5 and [^125^I]I-Z1. In combination with a higher brain-to-blood ratio of scFv8D3-fused affibodies, these results suggest that the fused affibodies did penetrate the BBB and bind to an intrabrain target. In contrast, [^125^I]I-Z5 and [^125^I]I-Z1 may have been associated with the endothelium of the brain vasculature at the early time point but without actually entering the brain.

Affibodies were generated and selected to bind Aβ_42CC_ and Aβ_42_wt protofibrils (39). Aβ selectivity was confirmed by *in vitro* autoradiography, where [^125^I]I-scFv8D3-Z5 discriminated tg-ArcSwe from WT in brain in regions commonly associated with Aβ-pathology. However, no difference was seen in whole brain, probably as a consequence of binding to TfR1, which is expressed on neurons throughout the brain. The ability of the affibodies to discriminate between WT and transgenic mice was assessed *in vivo* at 24 h after administration. [^125^I]I-Z5 and [^125^I]I-Z1 did not show greater retention in tg-ArcSwe or tg-Swe compared to WT mice at 24 h post injection. On the other hand, the scFv8D3-fused [^125^I]I-scFv8D3-Z1 and especially [^125^I]I-scFv8D3-Z5, showed a potentially higher retention in the transgenic group, although this trend was not significant. The intrinsic protofibril affinity may have been too low for the affibodies to achieve sufficient Aβ binding and retention *in vivo*, even with enhanced brain delivery. The Aβ affinity of the affibodies was 100-fold lower compared to di-scFv3D6-8D3; the latter has been shown, after radiolabeling, to be able to visualize Aβ pathology in PET 14 h after administration (33). In the nuclear track emulsion images, [^125^I]I-di-scFv8D3-8D3 appeared to accumulate around Aβ plaques, indicating Aβ specific binding and allowing for high contrast imaging, whereas the affibodies in the present study would likely not be able to visualize Aβ *in vivo*. As opposed to therapy, imaging applications rely on administration of low tracer doses of the compound. Therefore, high affinity to the intrabrain target is crucial for good imaging contrast. Comparing the 24 h brain retention, the scFv8D3 fused affibodies were similar to di-scFv3D6-8D3 in WT mice, but not in transgenic mice. A second explanation to the low ability of scFv8D3-fused affibodies to discriminate between WT and transgenic mice is the relation between TfR1 and Aβ affinity. Di-scFv3D6-8D3 displayed a low nanomolar TfR1 affinity in combination with picomolar Aβ affinity. For scFv8D3-fused affibodies, the TfR1-Aβ affinity relation was the opposite, with picomolar TfR1 affinity and nanomolar Aβ affinity. It is therefore likely that once inside the brain, these affibodies will preferentially bind to neuronal TfR1, which is equally expressed in the brain of WT and transgenic mice (9). Although Aβ binding sites in the transgenic mouse brain may be greater in number compared to TfR1, it is likely that too large differences between TfR1 and Aβ affinity will still favor TfR1 binding. To our knowledge, the optimal Aβ affinity range for BBB-penetrating biologicals has not been studied. A possibility to increasing the affinity is to use affibodies linked together as dimers to increase avidity, or through affinity maturation (39, 54).

## CONCLUSION

Affibody-scFv8D3 fusions showed higher brain uptake, and parenchymal delivery, compared to non-fused affibodies. The affibodies displayed tenfold higher total brain concentrations compared to regular antibodies. However, a suboptimal affinity towards Aβ in combination with high TfR1 affinity likely resulted in low target related brain retention in transgenic mice (tg-Swe and tg-ArcSwe) at 24 h after administration.

## Supplementary Information

Below is the link to the electronic supplementary material.

Supplementary file1 (PDF 1798 kb)

## ABBREVIATIONS

Aβ: Amyloid Beta
ABD: Albumin binding domain
AD: Alzheimer’s Disease
AUC_0-t_: Area under the curve from 0 to t
BBB: Blood–brain barrier
BCSFB: Blood-cerebrospinal fluid barrier
BCECs: Brain capillary endothelial cells
CNS: Central nervous system
CSF: Cerebrospinal fluid
ISF: Insterstitial fluid
scFv: Single chain variable fragment
RMT: Receptor Mediated Transcytosis
ROI: Region of interest
TfR1: Transferrin receptor 1
mTfR1: Murine transferrin receptor 1
Tg: Transgenic mice
Tg-ArcSwe: Tg-Swe AβPP transgenic mice with the Arctic and/or the Swedish AβPP mutations
WT: Wild type
