# Supplementary material for: Transferrin Receptor Binding BBB-Shuttle Facilitates Brain Delivery of Anti-Aβ-Affibodies
Source: Pharm Res. 2022 May 10;39(7):1509–21. doi: 10.1007/s11095-022-03282-2 (PMC9246779; doi:10.1007/s11095-022-03282-2)
Supplement: Supplementary file 1 — Supplementary file1 (PDF 1798 kb) [file 11095_2022_3282_MOESM1_ESM.pdf]

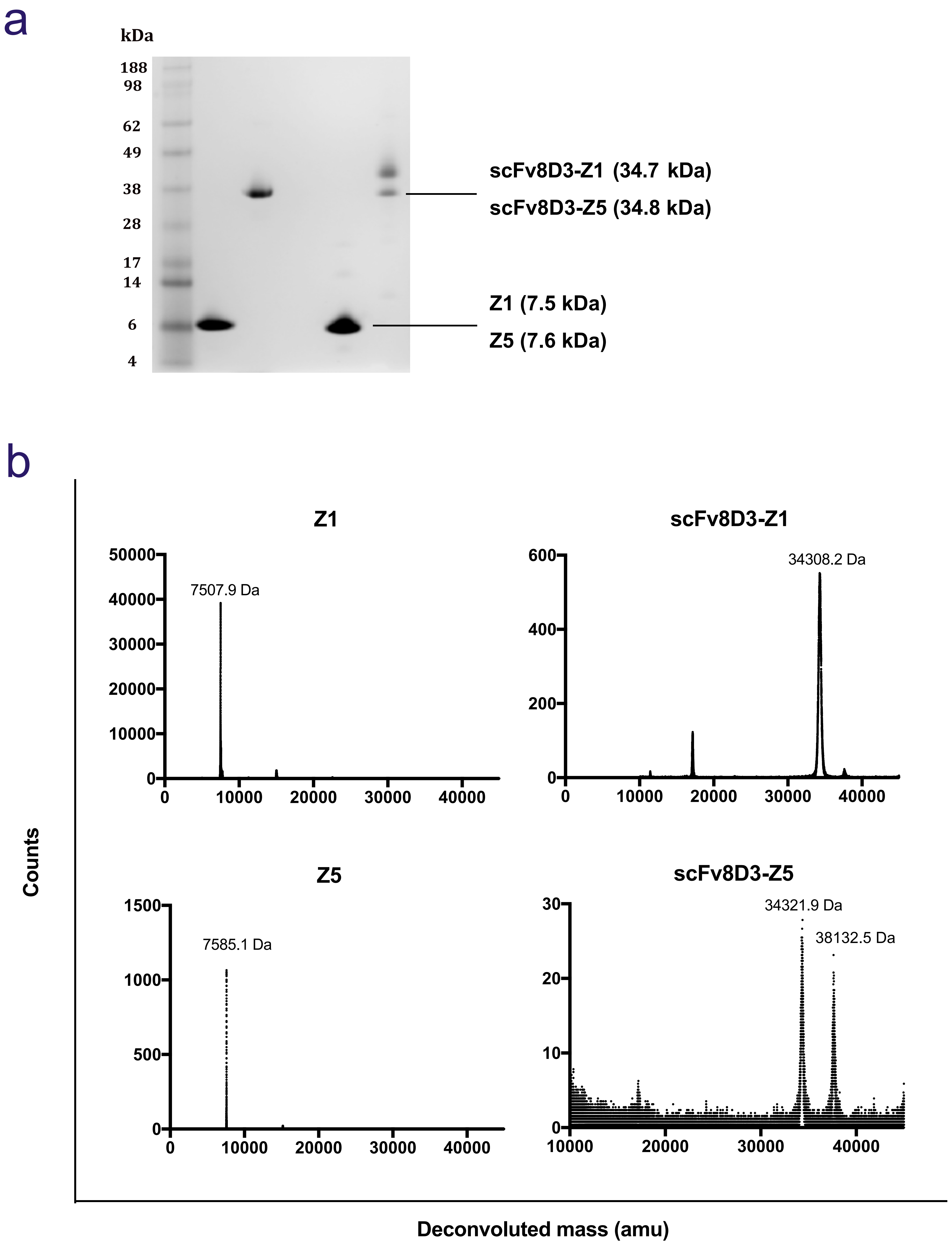

Supplementary Figure 1. (a) SDS-PAGE analysis of purified Affibody proteins. Marker, Z1, scFv8D3-Z1, Z5 and scFv8D3-Z5. Z1, scFv8D3-Z1, and Z5 appear as single bands of correct sizes of 7.5 kDa, 34.7 kDa and 7.6 kDa, respectively. The lane with scFv8D3-Z5 demonstrates two bands, including one of the expected size of 34.8 kDa and one of approximately 38 kDa. (b) MS MALDI-TOF spectra of the purified proteins. The identified masses of Z1, scFv8D3-Z1 and Z5 correspond to the theoretical masses of 7530 Da, 34737 Da and 7600 Da, respectively. MS spectrum for scFv8D3-Z5 shows two peaks, of which one corresponds to the expected mass of 34816 Da whereas the other peak measures an additional 4000 Da.
